# First detection of dengue virus in the saliva of immunocompetent murine model

**DOI:** 10.1590/0074-02760170208

**Published:** 2018-02-05

**Authors:** Arthur da Costa Rasinhas, Marcos Alexandre Nunes da Silva, Gabriela Cardoso Caldas, Fernanda Cunha Jácome, Raphael Leonardo, Flávia Barreto dos Santos, Priscila Conrado Guerra Nunes, Ortrud Monika Barth, Debora Ferreira Barreto-Vieira

**Affiliations:** 1 Fundação Oswaldo Cruz-Fiocruz, Instituto Oswaldo Cruz, Laboratório de Morfologia e Morfogênese Viral, Rio de Janeiro, RJ, Brasil; 2 Fundação Oswaldo Cruz-Fiocruz, Instituto Oswaldo Cruz, Laboratório de Imunologia Viral, Rio de Janeiro, RJ, Brasil

**Keywords:** dengue 4, saliva, BALB/c mice, immunocompetent murine model

## Abstract

The lack of an experimental animal model for the study of dengue pathogenesis is a limiting factor for the development of vaccines and drugs. In previous studies, our group demonstrated the susceptibility of BALB/c mice to infection by dengue virus (DENV) 1 and 2, and the virus was successfully isolated in several organs. In this study, BALB/c mice were experimentally infected intravenously with DENV-4, and samples of their saliva were collected. Viral RNA extracted from the saliva samples was subjected to qRT-PCR, with a detection limit of 0.002 PFU/mL. The presence of DENV-4 viral RNA was detected in the saliva of two mice, presenting viral titers of 10^9^ RNA/mL. The detection of DENV RNA via saliva sampling is not a common practice in dengue diagnosis, due to the lower detection rates in human patients. However, the results observed in this study seem to indicate that, as in humans, detection rates of DENV RNA in mouse saliva are also low, correlating the infection in both cases. This study reports the first DENV detection in the saliva of BALB/c immunocompetent mice experimentally infected with non-neuroadapted DENV-4.

Dengue (DEN) is an emerging disease, prevailing in urban and suburban areas of tropical and subtropical countries. World Health Organization (WHO) data show that annually at least 100 million infections occur in over 100 countries in which the disease is endemic. Other sources suggest that worldwide this number could be almost four-fold higher, closer to 390 million infections per year (Bhatt et al. 2013).

Classified as an arbovirus (arthropod-borne virus), the DEN virus (DENV) is a member of the *Flaviviridae* family, genus *Flavivirus*, and can be discriminated into four antigenically distinct serotypes: DENV serotype 1 (DENV-1), DENV-2, DENV-3, and DENV-4 (Reiner et al. 2016, WHO 2016). The virus is transmitted by the bite of *Aedes aegypti* or *Ae. albopictus* mosquitoes. Successful infection results in DEN (San Martín et al. 2010).

DENV is a spherical particle measuring approximately 40-60 nm in diameter, with a lipid envelope and icosahedral nucleocapsid that measures about 30 nm (Barth 2010). The viral genome comprises a single stranded, positive polarity RNA molecule, which is approximately 11 kilobases (kb) in length. The genome codifies three structural proteins, those of the capsid (C), the membrane (M) and the envelope (E), and seven non-structural proteins: NS-1, NS-2A, NS-2B, NS-3, NS-4A, NS-4B, and NS-5 (Guzman et al. 2010, Yamashita et al. 2016).

Despite being the only natural vertebrate hosts for DENV, non-human primates are not preferred as an animal model for experimental DEN infection, failing to show signs of the disease as observed in humans (Clark et al. 2013). The absence of a suitable animal model that successfully replicates the disease as it occurs naturally not only hampers the development of efficient vaccines and therapeutics, but also hinders a better understanding of the viral mechanisms of immunopathogenesis (Oliveira et al. 2016). Although some DENV strains induce limited viremia in some mouse strains, the overwhelming majority of immunocompetent mouse models do not present with clinical signs of DENV infection (Sarathy et al. 2015). Our group verified the susceptibility of immunocompetent BALB/c mice when infected by the intraperitoneal and intravenous routes with DENV nonneuroadapted viral strains. Focal alterations in the lung, heart, kidney, and hepatic tissue have been demonstrated (Paes et al. 2005, Barreto et al. 2007, Barreto-Vieira et al. 2015, Jácome et al. 2015). The virus particles were isolated in the *Ae. albopictus* C6/36 cell line inoculated with the supernatant of a macerate of the lung, cerebellum, kidney, and liver of infected animals. Viral antigen was detected in liver endothelial cells and in hepatocytes (Paes et al. 2005). A peak in viremia was detected on the 7th day post-infection (Paes et al. 2005).

Thus far, there have been no reports regarding the detection of DENV in the saliva of a DENV animal model. However, the virus has been detected in the saliva of infected human patients (Cuzzubbo et al. 1998, Balmaseda et al. 2003, Poloni et al. 2010, Yap et al. 2011, Anders et al. 2012, Andries et al. 2015).

The aim of the present study was to detect DENV in the saliva of an immunocompetent animal model, more specifically, BALB/c strain mice experimentally infected with DENV-4 for a better understanding of the pathogenesis of this virus.

*Ethical statement* - The Animal Ethics Committee (protocol LW-50/11) of Fundação Oswaldo Cruz (Fiocruz) approved the procedures performed in this study.

*Viral strain* - A DENV-4 strain was isolated in Rio de Janeiro, Brazil, from a DEN-positive patient identified in 2013. The serotype was confirmed by indirect immunofluorescence using DENV-4 specific monoclonal antibody and real-time quantitative polymerase chain reaction (RT-PCR) by the Flavivirus Laboratory, Instituto Oswaldo Cruz (IOC), Fiocruz. The viruses did not undergo any passage through mice brains, avoiding neuroadaptation.

*Production of the viral stock* - An aliquot of the viral strain was inoculated in a C6/36 cell culture (5 × 10^5^ cells/mL). The titration was performed according to Reed & Muench (1938). After three cell passages, the strain presented a viral titer of 10^9^ TCID_50_/mL and was selected for the experimental infection.

*Animals* - Male, 2-month-old BALB/c mice weighing between 20 and 25 g were obtained from the Instituto de Ciência e Tecnologia em Biomodelos (ICTB), Fiocruz. During the experimentation period, the mice were kept in ventilated shelves located in the vivarium of the Hélio e Peggy Pereira pavilion, where conditions such as temperature, humidity, feeding, ventilation, hygiene, and photoperiods were properly controlled. A total of 30 animals were used in the study. Fifteen animals were infected with DENV-4, and 15 were not infected and used as negative controls.

*Experimental infection* - A dose of 100 μL of the inoculum (presenting a viral titer of 10.000 TCID_50_/0.1 mL) was inoculated via the caudal vein. Mice of the control group were inoculated with 100 μL of Leibovitz medium (Sigma, Germany) via the caudal vein. The entire process was performed inside a biosafety cabinet.

*Saliva sampling* - The saliva sampling was performed 72 h post-infection. The mice were carefully physically restrained and a swab moistened with L-15 medium was gently inserted into the mouth of each mouse for 1 min. Afterwards, the swab containing the saliva sample was properly stored in a sterile microtube filled with 0.5 mL of L-15 medium and transferred to a -70°C freezer, for proper storage and subsequent molecular analysis. Only one sample was obtained from each animal.

*RNA extraction* - Viral RNA was extracted from 140 µL of a saliva sample using the QIAmp Viral RNA mini kit (Qiagen, Germany) according to the protocol described by the manufacturer. Negative controls were used during the entire procedure to ensure no cross contamination occurred among the samples. A positive DENV-4 sample was extracted and used only to test the protocol prior to sample testing to avoid cross contamination.

*RT-PCR* - For the detection and quantification of the viral RNA, a standard curve was prepared from the results obtained for serial dilutions of RNA extracted from a sample of DENV-4 of known titer (1.12 × 10^6^ TCID/0.1 mL).

Real-time reverse transcriptase PCR (TaqMan) assay (qRT-PCR) was used, as described by Johnson et al. (2005), with slight modifications described in Table. The primers were designed to anneal to the DENV-4 genome at position 904 (forward), 922 (reverse) and 960 (probe). According to the protocol, a positive result is considered up to a cycle threshold value of 36. Reverse transcription occurred at 50°C for 15 min, followed by 2 min at 95°C and 40 cycles of denaturation and amplification (95°C for 15 s; 60°C for 1 min).

**TABLE:** Termocycling parameters for the real-time reverse transcriptase polymerase chain reaction (qRT-PCR)

Stage	Temperature	Duration	N° of cycles
Reverse transcription	50°C	15 min	1
Enzyme activation	95°C	2 min	1
Denaturation	95°C	15 s	40
Hybridisation/extension	60°C	1 min	40

The protocol described by Johnson et al. (2005) was used for both viral RNA detection and quantification. A standard curve was established by using serial dilutions of a DENV-4 extracted with known titer (1.12 × 10^6^ TCID/0.1 mL). The assay was performed using the commercial SuperScript III Platinum One-Step Quantitative RT-PCR kit (Invitrogen Corporation, USA) and the following primers and probe: DENJ-4R (5′TCCACCTGAGACTCCTTCCA3′), DENJ-4F (5′TTGTCCTAATGATGCTGGTCG3′) and DENJ-4P (6-FAM 5′TTCCTACTCCTACGCATCGATTCCG3′ BHQ-1). The mixture (final volume of 20 µL) was prepared with 1 µL of each primer at 50 µM, 5 µL of a 2× reaction mixture (0.4 µM of each dNTP and 6 µM of MgSO_4_), 0.5 µL of SuperScript III reverse transcriptase and 3.5 µL of DNase/RNase free water, 1 µL of 5 mM MgSO_4_ and 0.75 µL of the probe (9 µM) and loaded into a 96-microwell optical microplate (PE Applied Biosystems, USA). Five microliters of the extracted RNA was added and the reaction was carried out in a LineGene 9660 thermocycler (Bioer, China).

During the entire experimentation period, none of the mice died as a result of infection. Some of the animals presented signs of hyperthermia, as observed by measuring body temperature, but other signs such as tremor, petechial, diarrhea, and neurological alterations were not observed.

The results of qRT-PCR (Figure) showed that the viral RNA was successfully detected in the saliva of two mice, from a total of 15 analysed samples. The viral titers observed in both samples reached an order of magnitude of 10^9^ (5.76 × 10^9^ and 8.41 × 10^9^ RNA copies/mL). Both animals displayed a slight increase of body temperature, reaching 37.5°C and 37.4°C, respectively.

**Figure:**
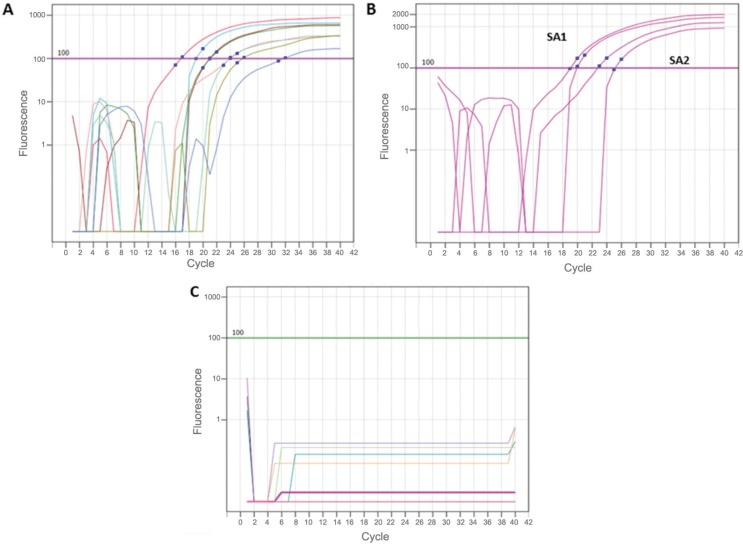
Real-time reverse transcriptase polymerase chain reaction (qRT-PCR) for detection and quantification of dengue virus-4 (DENV-4) in the saliva of experimentally infected BALB/c mice. (A) Standard curve constructed using five dilutions of the virus particles in duplicate. (B) Saliva samples (duplicates) from DENV-4 infected BALB/c mice. (C) Data from negative controls.

The present observation of DENV RNA in the saliva of mice are novel, yet the DENV RNA could be detected in human dengue cases, as previously demonstrated by Andries et al. (2015). In a group of 562 confirmed cases, 132 saliva samples showed the presence of the viral genome. Poloni et al. (2010) also reported the viral RNA in the saliva of two patients presenting symptoms of dengue, who were infected with DENV-2 and DENV-3. Several studies also showed the presence of DENV specific antibodies (IgA, IgG, and IgM) in the saliva of infected patients (Cuzzubbo et al. 1998, Balmaseda et al. 2003, Yap et al. 2011, Anders et al. 2012, Andries et al. 2015). The present study is the first report of the detection of DENV in the saliva of immunocompetent BALB/c mice experimentally infected with DENV-4. Further studies to determine the frequency of the DENV presence in the saliva of mice and to evaluate virus particle infectivity in this murine model are warranted. The authors agree that, in fact, this is a viral detection only, and no conclusions on viral infectivity and replication can be made using the current protocol.

*In conclusion* - Two of the analysed mice presented elevated titers of DENV-4 RNA copies in saliva samples, demonstrating that viral dissemination occurred. This fact further shows the susceptibility of the BALB/c strain to experimental infection with a non-neuroadapted DENV inoculum via a non-invasive route. Furthermore, the same mice also presented slight elevations of body temperature, which could be associated with infection. While these findings do not implicate saliva sampling as a good method for detecting the presence of DENV infection, they do correlate the detection of the virus in human saliva and mouse saliva, reiterating the similarities between the infection as observed in both the human and murine cases, as both demonstrate lower detection rates compared to the detection rate in other sample types, such as serum or tissue.
